# A Multi Targeting Conditionally Replicating Adenovirus Displays Enhanced Oncolysis while Maintaining Expression of Immunotherapeutic Agents

**DOI:** 10.1371/journal.pone.0145272

**Published:** 2015-12-21

**Authors:** G. Clement Dobbins, Hideyo Ugai, David T. Curiel, G. Yancey Gillespie

**Affiliations:** 1 Department of Pediatrics, University of Alabama at Birmingham, Birmingham, Alabama, United States of America; 2 Department of Neurosurgery, University of Alabama at Birmingham, Birmingham, Alabama, United States of America; 3 Cancer Biology Division, Department of Radiation Oncology, School of Medicine, Washington University in St. Louis, St. Louis, Missouri, United States of America; 4 Biologic Therapeutics Center, Department of Radiation Oncology, School of Medicine, Washington University in St. Louis, Missouri, United States of America; Memrial University, CANADA

## Abstract

Studies have demonstrated that oncolytic adenoviruses based on a 24 base pair deletion in the viral E1A gene (D24) may be promising therapeutics for treating a number of cancer types. In order to increase the therapeutic potential of these oncolytic viruses, a novel conditionally replicating adenovirus targeting multiple receptors upregulated on tumors was generated by incorporating an Ad5/3 fiber with a carboxyl terminus RGD ligand. The virus displayed full cytopathic effect in all tumor lines assayed at low titers with improved cytotoxicity over Ad5-RGD D24, Ad5/3 D24 and an HSV oncolytic virus. The virus was then engineered to deliver immunotherapeutic agents such as GM-CSF while maintaining enhanced heterogenic oncolysis.

## Introduction

Virotherapy uses human viruses to kill infected cancer cells by targeting the virus infection and replication to different cancer types. Initially, viral therapy for cancer treatment centered on infecting a small number of target cells with a replicating virus, which would replicate, amplify, and spread to adjacent cells, destroying the tumor by a lytic mechanism called oncolysis. Adenoviruses (Ad) have a number of advantages as an oncolytic virus (OV): they achieve a high rate of human cell transduction, can be grown and administered at high titers, and are clinically safe [1–3].

Oncolytic adenoviruses based on a 24-base pair deletion (D24) in the nucleotide sequence encoding the Rb-binding domain of the immediate early gene E1A, result in a virus deficient for viral replication in normal cells [4–7]. Importantly, the 24-base pair deletion does not compromise Ad replication efficiency in cancer cells or oncolytic potency [4].

However, first-generation conditionally replicating adenoviruses (CRAds) had limited efficacy as the therapeutics were based on the Ad5 serotype. The primary receptor for Ad5 is the coxsackievirus B and adenovirus receptor (CAR) which is poorly expressed on most cancer cells [8]. Furthermore, CAR is highly expressed on lung and liver cells severely limiting the potential of the virus [9, 10]. Although these CRAds lacked efficacy in failing to infect an adequate number of cancer cells, the therapeutics were proven safe for patient treatment [11].

Hence, our research has focused on overcoming the low level of cancer cell transduction. A number of strategies have been employed to improve first generation CRAd cancer targeting. One involves genetic incorporation of small peptide ligands with receptor targeting properties into the Ad fiber knob. Due in part to structural constraints, only a limited number of small peptide ligands can be used to produce functional fibers that enhance virus infectivity. One successful ligand is the RGD (Arg-Gly-Asp) motif which has high binding affinity to integrins such as αvβ3 and αvβ5, secondary Ad receptors that promote Ad internalization and are highly expressed on many cancer types. Importantly, incorporation of this ligand into the fiber knob did not result in loss of vector binding; and a CRAd, Ad5-RGD D24, based on this modification displayed dramatic enhancement in oncolysis of certain cancer lines with high expression of integrins and is in clinical trials (ClinicalTrials.gov identifier NCT00805376) [11].

A second approach has been to construct a recombinant chimera fiber with the knob domain replaced with that from another Ad serotype. As an important region responsible for binding to cell surface receptors, the fiber knob domain represents a major determinant of Ad tropism. For example, replacing the Ad5 knob with the Ad3 knob, resulting in Ad5/3 serotype chimera, has proven successful in re-targeting the vector to cells with low levels of CAR, but high levels of the Ad3 receptor(s) which are upregulated on a number of cancer cell types [5, 6, 12, 13].

This infectivity enhancement has become an area of research focus as Ad3 binding of the Desmoglein 2 (DSG2) receptor, a primary receptor for the Ad3 serotype, improves the tumor microenvironment for drug delivery as the interaction results in a signaling cascade that releases the tight epithelial adhesion that normally precludes white blood cell and therapeutic agent tumor penetration [14, 15]. The Ad5/3 modification serves a second important function by avoiding the coxsackievirus and adenovirus receptor that exists on many normal cells allowing improved tumor selection. Hence, the use of this virus has numerous potential therapeutic benefits. As with the Ad5-RGD D24 virus, the Ad5/3 D24 vector also exhibits a substantial improvement in the transduction efficiency of certain cancer cells [16].

Treatments based on oncolytic viruses have shown positive responses for some patients. However, evidence suggests that beneficial outcomes are most likely due to an immune response to the OV infected cancer cells and to a lesser extent direct oncolysis by the vector. A plausible explanation is that tumor associated antigens released by oncolysis were successfully processed by antigen presenting cells (potentially induced by the inflammatory response to “danger signals” provided by the pathogenic stimuli) [17]. The outcome being that T effector cells generated to viral antigens would, through cross-epitope spreading, recognize tumor antigens, overcome the immunosuppressive signals of the cancer, resulting in cancer elimination. Hence, increasing the proportion of CRAd-infected cancer cells should enhance such an outcome, as generating a more extensive debris field in a more heterogenic number of cancer cells may increase potential tumor specific antigens (TSAs). This has become an important consideration given that multiple molecular subtypes of tumor cells can be found within any one tumor mass, confounding the ability to target a specific tumor genotype [18]. With heterogenic oncolytic enhancement as the primary goal, this study first looked at augmenting second generation CRAds by incorporating D24, Ad5/3 serotype chimera and an RGD modified fiber into a single OV: Ad5/3-C-RGD D24.

The Ad5/3-C-RGD D24 genome was successfully engineered and rescued. The new CRAd was assayed for oncolysis in a number of cancer lines and displayed cytotoxicity for all lines assayed that was equal or improved when compared to an array of other oncolytic viruses. In order to aid a more consistent immune reaction to infected cancer cells, the CRAd was further engineered to ascertain if the virus could be an immunotherapeutic drug delivery and or potential vaccine platform while maintaining high heterogenic oncolysis.

## Materials and Methods

### Construction of the multi-targeting adenoviral plasmid DNAs

To generate the virus genomes a double homologous recombination strategy was employed between a modified AdEasy-1 (AdEz)-derived backbone vector and various shuttle vectors. The Ad5 vector, with deleted E1 and E3 regions of the Ad5 genome (AdEz), was modified by inserting a *SwaI* restriction enzyme site in place of the deleted fiber gene to yield AdEzDF, aiding recombination with shuttle vectors incorporating further changes in the Ad genome.

The shuttle vectors were of two variants: one contained the tail and shaft of the Ad5 serotype but the knob from Ad3. This vector has been further modified to contain the unique restriction enzyme sites *BamH1* and *MfeI* on the Carboxyl terminus (C-terminus) of the Ad3 knob domain for easy addition of motifs such as the 27 nucleotide (nt) long oligonucleotide duplex encoding CDCRGDCFC peptide (RGD) (Ad5/3-C-RGD) shuttle vector. These shuttle vectors have been engineered with flanking regions that contain homologous recombination (HR) arms with the AdEzDF genome. The second shuttle vector variant employed was the delta 24 E1 shuttle vector (D24), containing the 24 base pair deletion in the E1A gene [19].

**Fig 1:**
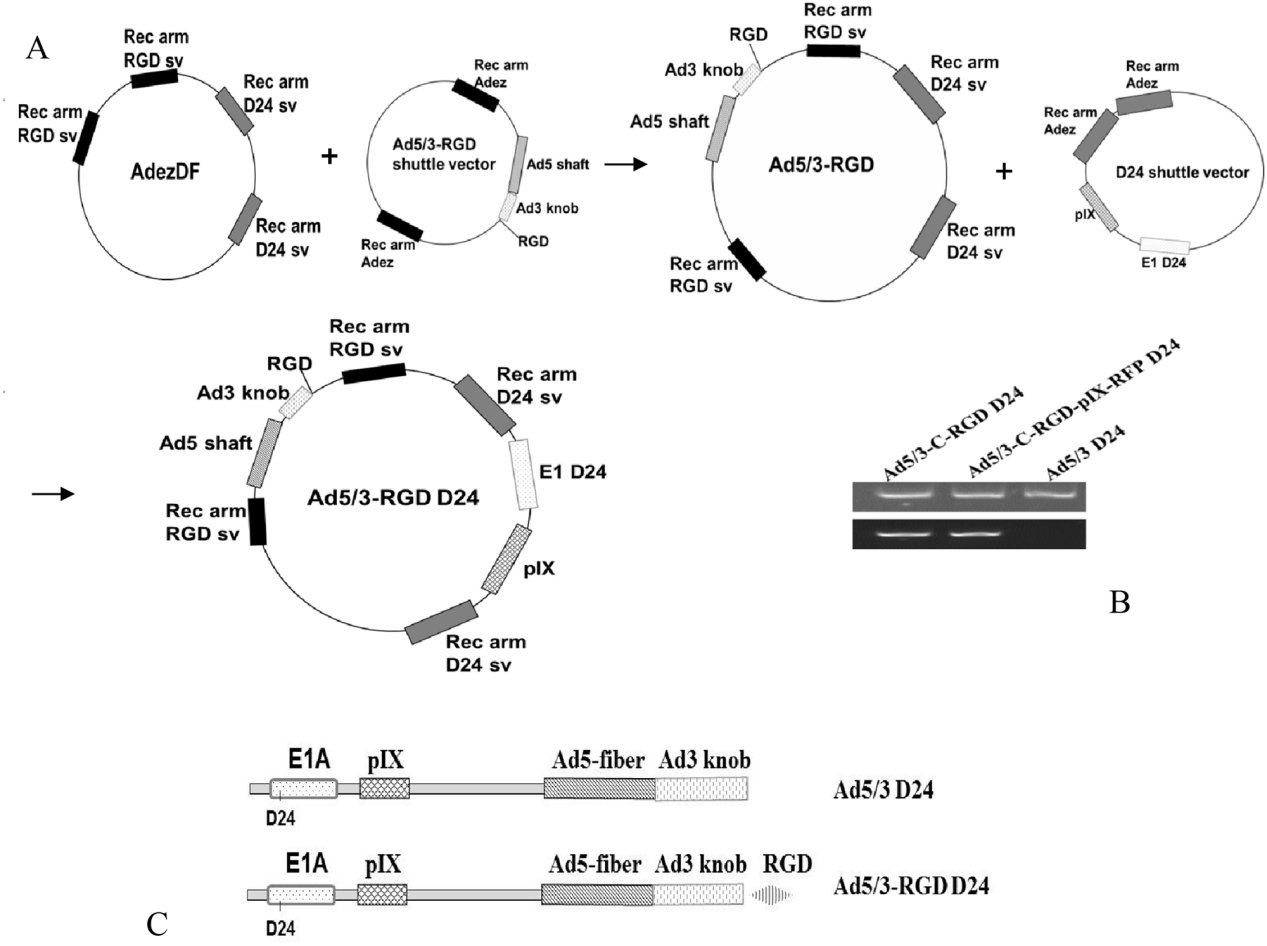
Virus construction. (A) The two step HR strategy employed to generate the Ad5/3-C-RGD D24 genome. (B) PCR of the fiber region using adenoviral DNA prepared from purified virus particles. Top panel represents PCR products amplified using specific primer sets for Ad3 knob region. Bottom panel represents PCR products amplified using specific primer sets for Ad3 knob region and RGD on Ad3 knob C-terminus. (C) Schematic representation of the single and multi-targeting viruses.

The first HR was carried out between the AdEzDF and the Ad5/3-C-RGD shuttle vector in the recombination-prone *Escherichia coli* (*E*. *coli*) strain BJ5183 [20]. This Ad5/3-C-RGD construct was then recombined with the D24 shuttle (Fig 1). The isogenic control Ad5/3 D24 was generated by recombining the AdEzDF with the unmodified Ad5/3 shuttle vector followed by recombination with the D24 shuttle vector. PCR and nucleotide sequencing were carried out on the viral particle genomes to ensure they were isogenic and that the relevant modifications were intact.

### Ad5/3-C-RGD D24 engineered for drug delivery

As a tumor associated antigen (TAA) or tumor specific antigen (TSA) vaccine proof of principle, a second variant of the D24 shuttle vector was generated. To clone potential TSAs onto the capsid of Ad5/3-RGD D24, the D24 shuttle vector containing the capsid protein pIX was engineered to contain unique *Nhe1* and *Sal1* sites on to the C-terminus of the protein allowing for the cloning of large genes into this region. As before the shuttle vector contains recombination arms that replaces full length E1 with an E1 containing the deleted 24 nucleotides. To assay the potential of the site as a vaccine approach and to ensure that the modification would be compatible with the existing multi-targeting genome modifications, the monomeric red fluorescent protein (RFP) was cloned into the C-terminus of pIX and homologously recombined as above. After screening the genome to ensure the relevant modifications were intact, the full length genomes were *Pac1* digested and transfected into 293 cells.

To further test the ability of the multi-targeting OV to deliver immunotherapeutic agents, a new virus was engineered to deliver immunostimulatory agents such as antibodies and cytokines. Construction of the new virus was initiated by engineering a number of modified shuttle vectors (SV). Similar to the one made above, these vectors contain recombination arms with AdezDF-*swa1*; and the fiber was modified in a similar way to incorporate the Ad5 shaft with the Ad3 serotype knob followed by the RGD modification on the C-terminus. A large portion of the E3 gene was deleted and replaced with unique restriction sites *BstBI* and *SalI* for cloning of immunostimulatory agents. The adenovirus death protein (ADP) was included in the shuttle vector N-terminus of the cloning. Other genes known to decrease the immune response were removed.

A number of SV modifications were tried until one was found that proved compatible with rescuing the virus. Double homologous recombination steps were carried out as before. Following the successful rescue of Ad5/3-RGD E3 D24, a new shuttle vector incorporating human GM-CSF was generated by amplifying the cytokine from pORF-hGMCSF (Invivogen, San Diego CA) and subcloning the immunostimulant into the unique *BstBI* and *SalI* sites, then recombined as above.

### Generation, Propagation, Purification, and Titration of Adenoviruses

Following molecular validation, adenoviral plasmid DNAs were digested with *PacI* and transfected into 293 cells, using Lipofectamine LTX (Invitrogen; Carlsbad, CA). The cells were harvested 2 weeks later when cytopathic effect (CPE) was observed and disrupted by four freeze/thaw cycles. Cell lysates were used to upscale the virus in A549 cells. The viruses were then purified by double cesium chloride (CsCl) density gradient centrifugation, dialyzed and stored at -80°C, as previously described [21]. DNA was extracted from purified virus particles using Qiagen QIAmp DNA mini kit (Qiagen, Valencia, CA); PCR and sequencing were used to verify that the relevant modifications were present. The infectious titer (plaque forming units [PFU]/ml) of the purified Ad vectors were determined by 50% Tissue Culture Infective Dose (TCID_50_) assay using A549 cells, as previously described [22]. The physical particle titer (VP/ml) was calculated based on the protein amount of the purified adenovirus determined at an absorbance of 260 nm (OD260) as described previously by Maizel *et al*. [23].

### Cell Lines

Human glioma U87, human lung epithelial A549, human breast epithelial ZR-75-1, human prostate epithelial DU145 and human foreskin fibroblast HFF cell lines were obtained from the American Type Culture Collection (ATCC; Manassas, VA). The human glioma lines A172, U105, U138 and U251 were from Dr. Darell D. Bigner [24] (Duke University Medical School; Durham, NC). The ovarian carcinoma line, OV-4 (ATCC), was from Dr. Timothy J. Eberlein (Brigham and Women’s Hospital, Harvard Medical School, Boston, MA.). The metastatic mouse breast cancer cell line 4T1 (ATCC) and pancreatic cell line BxPC3 (ATCC) were from Dr. Donald Buchsbaum (UAB); and the neuroblastoma cell lines SK-N-As (ATCC) and SK-N-Be (ATCC) were from Dr. Elizabeth Beierle (UAB). All lines were cultured in Dulbecco’s Modified Eagle’s Medium/Ham’s Nutrient Mixture F-12, (DMEM/F12; Sigma-Aldrich; St. Louis, MO) containing 10% fetal bovine serum, (FBS; Hyclone; Logan, UT), 2 mM L-glutamine, 100 U/ml penicillin (Mediatech, Inc., Herndon, VA).

### Flow Cytometry

Cell lines were detached from a 75-cm^2^ tissue culture flask by treatment with cell stripper (Cellgro; Manassas, VA.). Cells were counted, blocked and aliquots of 1.5 × 10^6^ cells were incubated with 1.5 μg of anti-αVβ3 and anti-αVβ5 integrin antibodies (Millipore; Temecula, CA), 1.5 μg of mouse monoclonal antibody Desmoglein 2 (DSG2) (clone AH12.2, Santa Cruz Biotechnology Inc, Santa Cruz, CA) or untreated (control). Following primary antibody incubation, the cells were washed and incubated with Alexa fluor 488-conjugated goat anti-mouse immunoglobulin G (Invitrogen) diluted in PBS with 0.1% BSA. Cells were then diluted in PBS and a cytometric analysis of 10,000 events per sample was conducted using FACScan with CellQuest software (Becton Dickinson, Mountain View, CA).

### Cell viability assays

Cells were plated at 10,000 cells per well on 96-well plates and infected the following day with viruses at 1–100 viral particles (VP)/cell or 0.1–1 PFU/cell in triplicate. Infections were carried out in 2% FBS. Two to ten days post infection, depending on when full CPE was first visually detected, cell viability was analyzed via MTS assay (Cell Titer 96 AQueous One Solution Cell Proliferation Assay, Promega; Madison, WI). Cell killing activity was measured relative to percentage of uninfected cells. Data are presented as mean ± standard deviation. The titers for the OVs used in the study are in S1 and S2 Tables. Comparisons between Ad viruses were based on physical particle titer (VP/cell) as the measurement is more consistent between laboratories [23] and includes total viral particles which may be clinically relevant. Infectious titer (PFU/cell) was used for comparisons with the HSV virus; as an enveloped virus, OD260 measurements are not reliable.

### Visualization and expression

For phase contrast and fluorescent live images, cells were grown and infected as in the viability assays and imaged with a Nikon inverted microscope equipped with a Nikon DS FI1 camera (Nikon Instruments Inc., Melville, NY) and analyzed as previously described [25]. For high magnification images, cells were grown in Lab Tek II chamber slides (Nunc; Rochester, NY) in the same conditions as viability assays. On the indicated days, cells were fixed with 4% formaldehyde, washed with PBS and mounted using Vectashield mounting medium containing DAPI (Vector Laboratories; Burlingame, CA). Images were overlaid with NIS-Element ARTM software.

For measuring GM-CSF production, cells were grown as for the MTS assays and infected with virus at the indicated titers. Medium was harvested on the indicated days and measured via ELISA (Biolegend; San Diego, CA).

### Multi-Step Curve analysis

Sk N-As and Sk N-Be cell lines were grown as above in 6 well plates and infected with adenovirus at an MOI of 1 PFU/cell. Culture medium containing the virus released from the infected cells was arvested at 24, 48, 72 and 96 hours post infection. Infectious titers from the medium were measured via TCID50 on 549 cells.

## Results

### Third generation multi-targeting CRAd construction and generation

Construction of the multi-targeting virus was carried out using standard cloning techniques described in the Materials and Methods section. A schematic representation of plasmid DNAs, genome construction and the oncolytic adenoviruses generated are shown in Fig 1. Ad5/3-C-RGD D24 (Ad5/3-RGD D24) and an isogenic Ad5/3 D24 control were rescued, grown to large scale, and purified by ultracentrifugation. DNA from purified virus particles was amplified by PCR using specific primer sets to confirm RGD insertion in the carboxy terminus of the Ad3 knob (Fig 1B). Subsequently, the nucleotide sequence of the Ad3 knob region and the D24 region for each virus was verified by sequencing.

### Incorporation of an RGD motif in the C-terminus of the Ad3 knob domain enhances oncolysis

**Fig 2:**
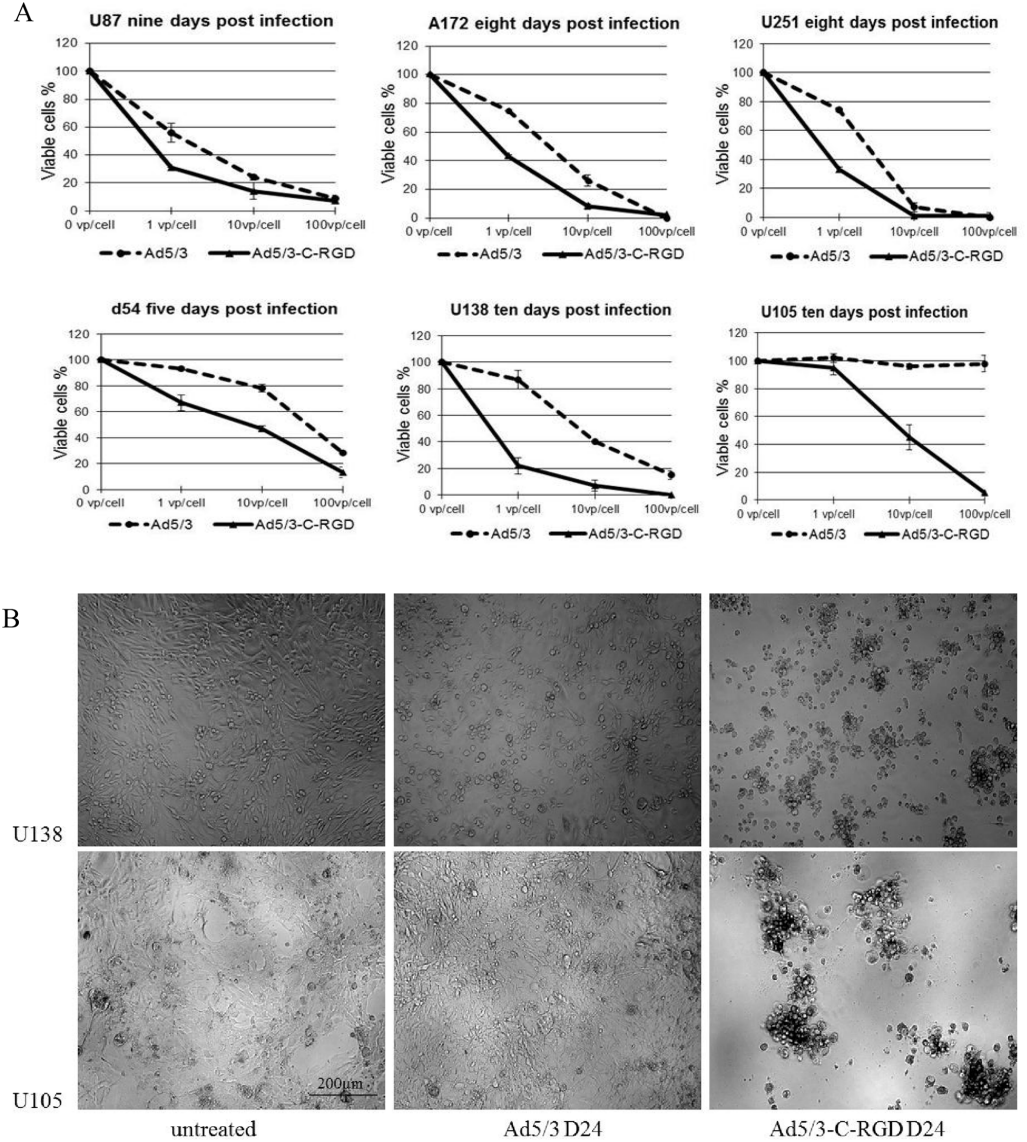
Cell killing assays in glioma cell lines. (A) Ad5/3 D24 and Ad5/3-C-RGD D24 were compared in the glioma lines U87, A172, U251, D54 U138 and U105. Cell viability was measured by MTS assay on the days indicated. All experiments were carried out in triplicate, three or more times. Error bars represent standard deviations. (B) Phase contrast images of U105 cells treated in same conditions as MTS assays nine days post infection with Ad5/3 D24 or Ad5/3-C-RGD D24 at an MOI of 100 VP/cell (100 × magnification live images in a 96 well).

Considering that Ad5/3 D24 virus shows promise in treating a range of cancer types and has potential for drug delivery [16], an analysis of infection and oncolysis was carried out to determine if the Ad5/3-C-RGD D24 multi-targeting strategy improved oncolysis over the second generation CRAd. Work first began on analyzing CPE of the OVs in glioma lines, as these tumors are one of the deadliest cancers, are known to have reduced CAR expression [8], and a number of oncolytic viruses have been tested for treatment of the disease. Similar to previous reports [26], initial studies showed that the Ad5/3 component enhanced cancer killing over the first generation Ad5 D24 virus (S1 Fig). Interestingly, the Ad5/3-C-RGD D24 showed enhancement in glioma killing in all lines assayed. In U251, A172, and U87, Ad5/3-C-RGD D24 showed improvement in killing potency over Ad5/3 D24 at low titer levels. By 10 days post infection with Ad5/3-C-RGD D24 (MOI 100 VP/cell) only minimal cell survival was detected. The cell lines with the greatest variability between the oncolytic viruses were the U138 and U105 cell lines, with Ad5/3 D24 displaying little to no cell killing 10 days post infection in the U105 line (Fig 2A). Phase contrast images show a consistent monolayer of U105 and U138 cells in the Ad5/3 D24 wells similar to untreated wells at 10 days post infection compared to full cytopathic effect seen in the Ad5/3-C-RGD D24 treated wells (Fig 2B).

Although D24 viruses are safe in patients, there is evidence of limited replication in normal cells [27–29]. However, the multi-targeting modifications did not result in an increase in cell killing over the Ad5/3 D24 virus in a normal cell line (S2 Fig).

**Fig 3:**
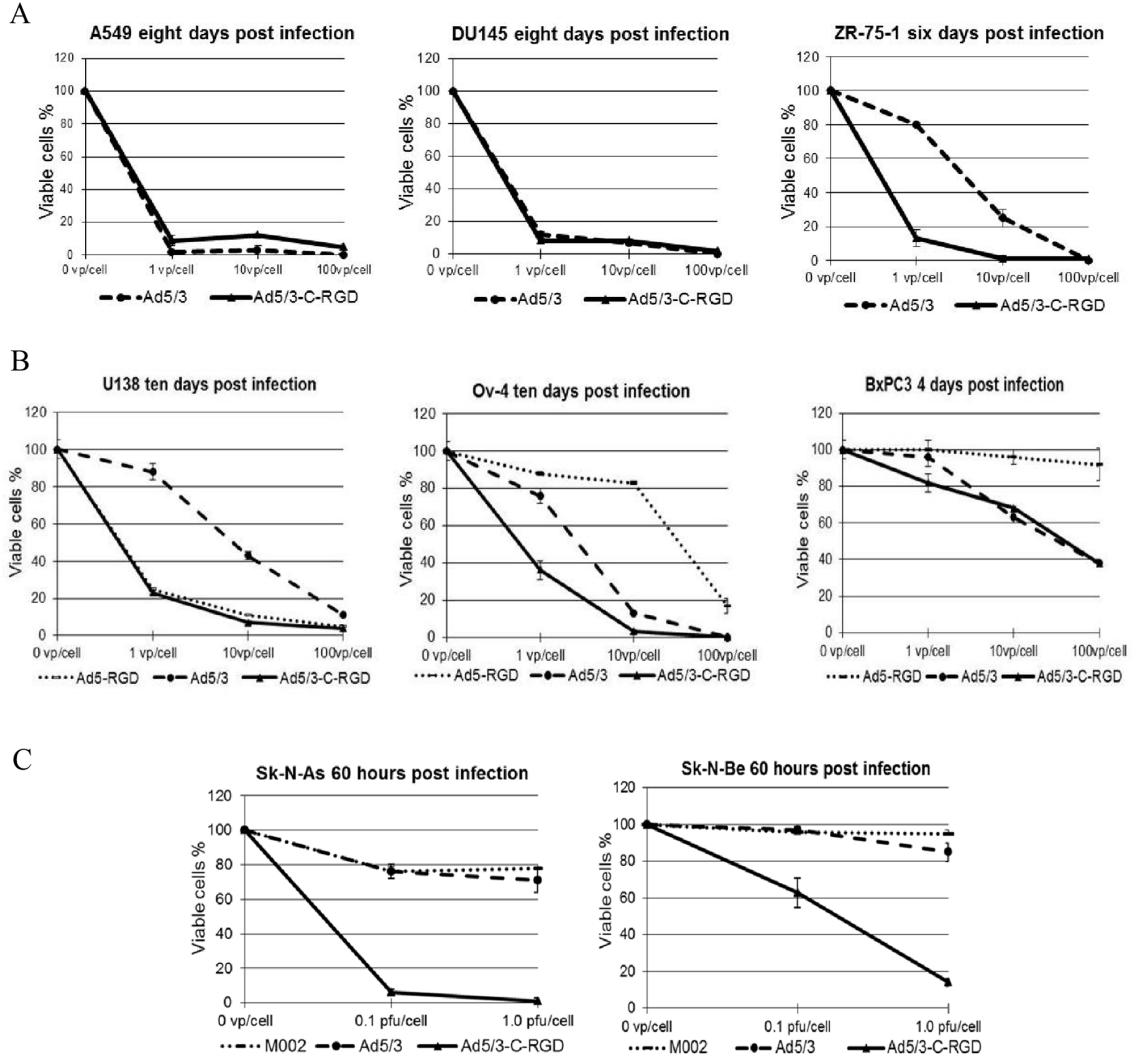
Cell killing assays in other cancer lines with a range of oncolytic viruses. (A) Further cell viability assays in the cancer lines A549 (lung), DU145 (prostate), and ZR-75-1 (breast). (B) Cancer killing in glioma, ovarian and pancreatic lines of the second generation OVs Ad5-RGD D24, Ad5/3 D24, and the third generation Ad5/3-C-RGD D24. (C) Cancer killing in two neuroblastoma lines by the indicated OVs.

Given the enhancement in cytotoxicity in the glioma lines, the viruses were assayed in three other cancer lines: lung (A549), prostate (DU145), and breast (ZR-75-1). The Ad5/3-C-RGD D24 showed similar oncolytic effect to Ad5/3 D24 in the lung and prostate lines. However, in the breast cancer line, an improvement in cell killing was again detected with the RGD modified virus (Fig 3A and S3 Fig).

Based on the improvement in cancer killing with the multi-targeting approach, analysis has begun on a comparison with other promising OVs, the Ad5-RGD D24 [7] and a herpes simplex virus (HSV) oncolytic virus [30]. Although no enhancement was seen in the glioma lines over Ad5-RGD D24, the multi-targeting virus was enhanced in the pancreatic and ovarian lines (Fig 3B). A comparison with the HSV OV M002 was carried out as well. As the M002 virus is effective in animal models of neuroblastoma [31], the Ad5/3-C-RGD D24 was assayed in two neuroblastoma lines. In both lines the multi-targeting CRAd displayed efficient release into the culture medium as determined by TCID50 (S4 Fig). Moreover, Ad5/3-C-RGD D24 showed enhancement in tumor cell killing 60 hours post infection over the HSV virus and Ad5/3 D24 virus (Fig 3C and S5 Fig). Further analysis comparing the third generation Ad5/3-C-RGD D24 virus to the second generation oncolytic viruses is continuing; however, the evidence is strong that the multi-targeting strategy is equal or superior at tumor cell killing in vitro. To date, this virus has infected and killed all cancer cell lines assayed at 1 PFU/cell or lower.

**Fig 4:**
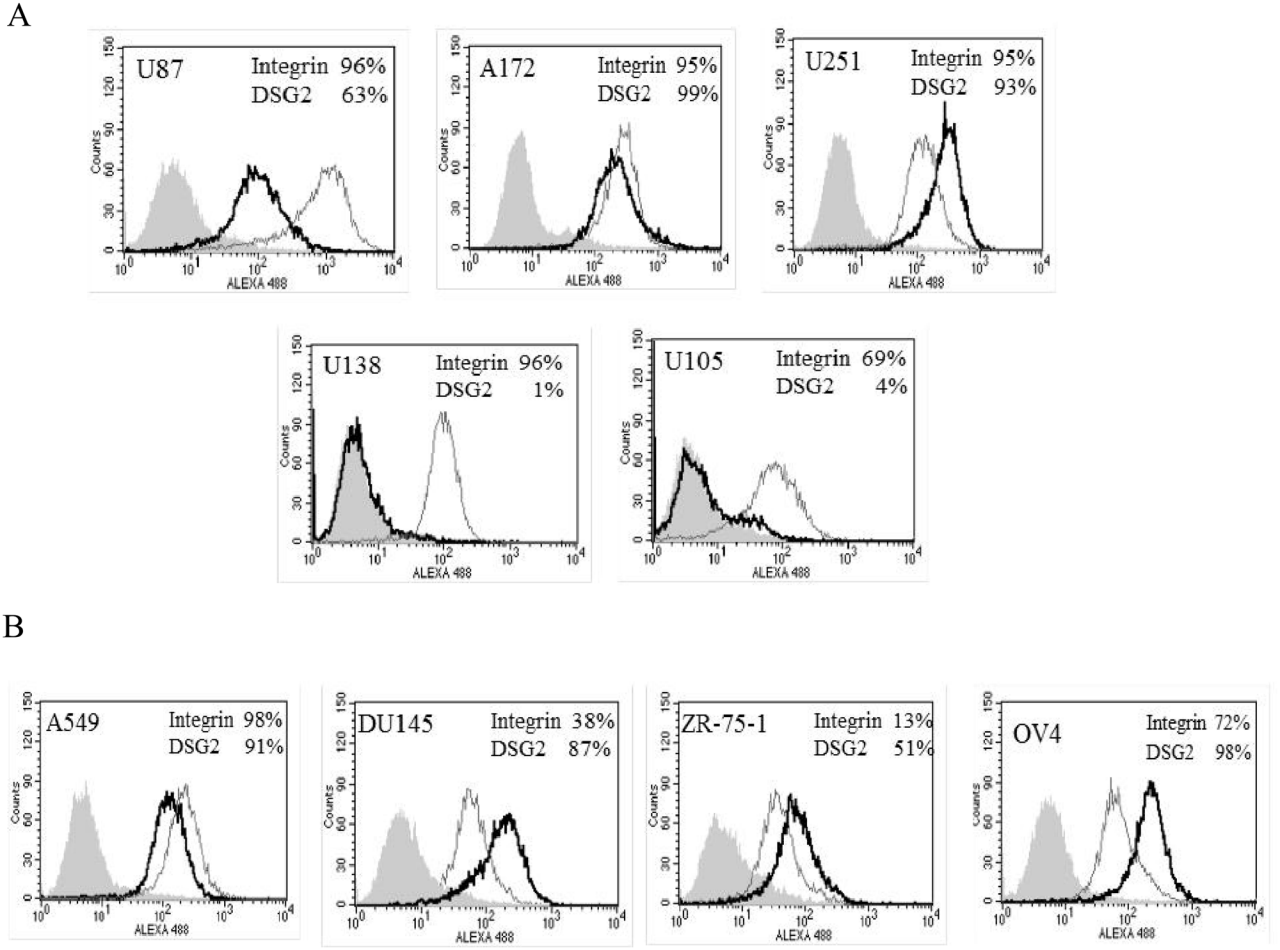
Integrin and DSG2 expression. (A) Analysis of integrin and DSG2 expression was carried out via flow cytometry in the indicated glioma lines and (B) other cancer lines. Filled grey histograms indicate unstained control cells; open black histograms indicate DSG2; open grey histograms indicate integrin stained cells. Percentages of positively stained cells are also illustrated.

As expected the enhanced oncolysis is related to the receptors being expressed on the cells. Flow cytometry (FCM) has been performed on a number of the cancer lines. FCM for αVβ3 and αVβ5 integrin (RGD receptors) expression confirmed that all glioma lines expressed integrins. Analysis of a primary receptor for Ad5/3, Desmoglein 2 (DSG2), indicated that the receptor was expressed in U87, A172, and U251 cell lines; however, little to no levels of the receptor could be detected in the U138 and U105 glioma lines (Fig 4A). FCM analysis of the lung (A549), prostate (DU145), breast (ZR75-1) and ovarian (OV4) indicated that integrin and DSG2 were present (Fig 4B). The Ad5/3 D24 showed similar killing to the multi-targeting virus in cells that expressed high levels of DSG2 such as the lung and prostate cancer cell lines. However, in the U138 lines and the U105 lines, DSG2 is expressed at low levels; and the virus showed attenuated cell killing compared to the multi-targeting virus. Interestingly, in some lines such as the breast and ovarian lines DSG2 is expressed; however, Ad5/3-C-RGD D24 showed superior killing at low titers.

### Ad5/3-C-RGD D24 engineered for immunotherapy

As the primary focus for virotherapy now is to generate an immune response against the infected cancer cells, this multi-targeting virus is a promising new tool as it should infect a greater and more heterogenic number of cancer cells potentially augmenting the T-effector cell response. To further evaluate Ad5/3-C-RGD D24 as an immunotherapeutic platform, the virus was first tested for potential delivery of neoantigens in a vaccine strategy. With the advent of inexpensive next generation sequencing and other techniques, there is now the possibility of isolating mutations in the exome of a tumor, potentially predicting TSAs that could be used as a future vaccine. The success of a vaccine depends on not just tumor-rejection antigens, but also immune modulators and adjuvants to stimulate a potent immune response. Inherently, Ads are both strong immune modulators and adjuvants as the virus initiates an innate immune response via Ad DNA/capsid proteins as well as an adaptive response from both Ad-specific CD4+ and CD8+ T cells [32]. Hence, capsid proteins such as pIX are promising regions in which to clone specific tumor antigens. Previous studies have shown that pIX can tolerate C-terminal fusions with substantially larger ligands than the fiber portion of the virus [33]. Given that Ad5/3-C-RGD D24 can infect a wide array of cancer cells at low titers the capsid of the virus is a promising platform to attach neoantigens.

**Fig 5:**
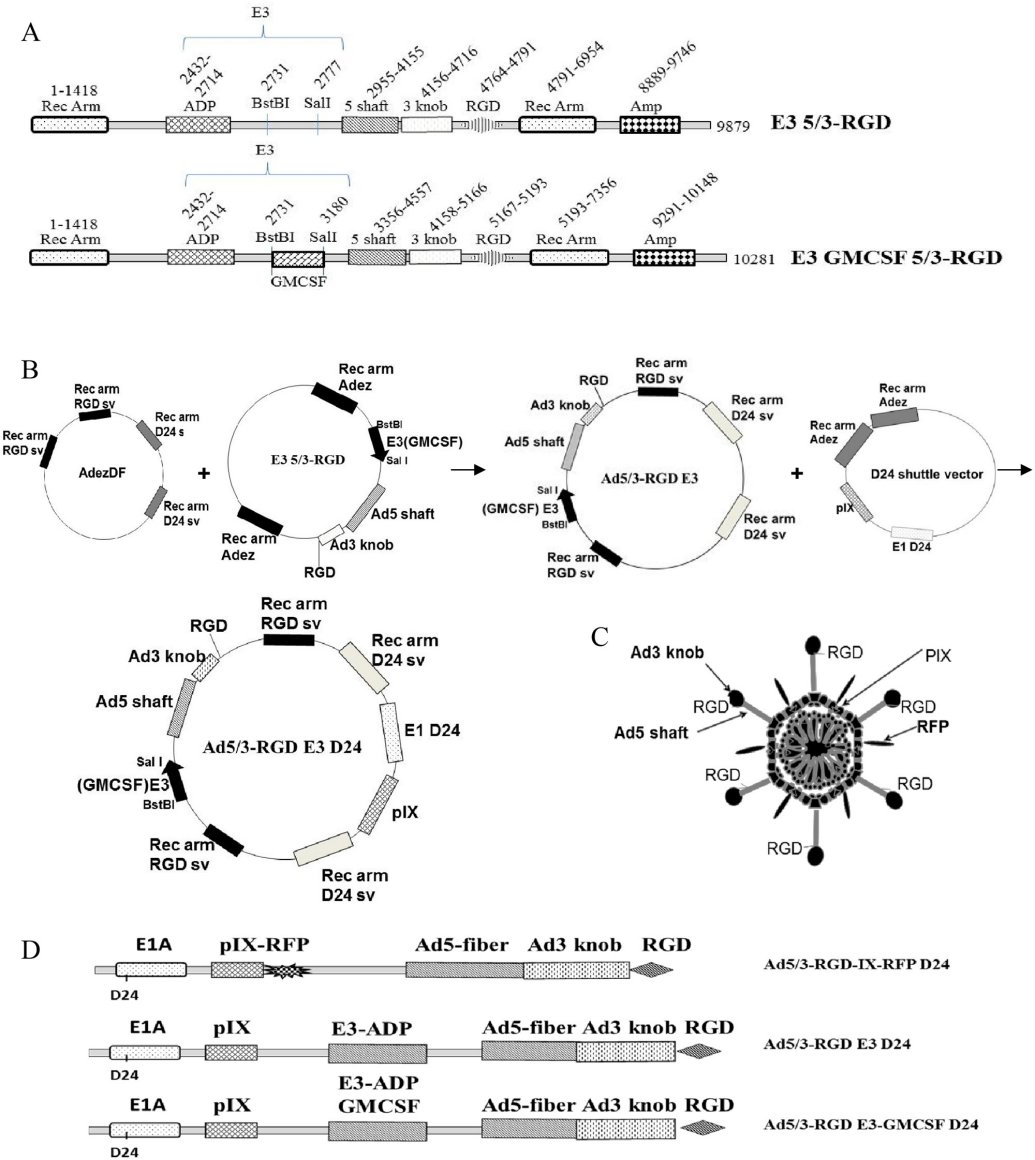
Construction of Ad5/3-C-RGD D24 immunotherapy platform. (A) E3 shuttle vectors generated for drug delivery. (B) Two-step homologous recombination to generate the various immunotherapeutic delivery OVs. (C) Illustration of the Ad5/3-RGD-IX-RFP D24 virus. (D) Schematic representation of the modified Ad5/3-C-RGD D24 CRAds.

As a potential vaccine proof of principle, a second variant of the D24 shuttle vector was generated containing the unique *Nhe1* and *Sal1* sites on to the C-terminus of the protein allowing for the cloning of large genes into this region. As before the shuttle vector contains recombination arms that replaces full length E1 with an E1 containing the deleted 24 nucleotides. To assay the potential of the site in a vaccine approach and to ensure that the modification would be compatible with the existing multi-targeting genome modifications, the monomeric red fluorescent protein (RFP) was cloned onto the C-terminus of pIX. Such a modification will also aid in tracking the virus. Shuttle vectors were then generated and recombined as above (Fig 5). After screening the genome to ensure the relevant modifications were intact, the full length genomes were *PacI* digested and transfected into 293 cells. Upon plaque formation and fluorescence detection, the Ad5/3-RGD-IX-RFP D24 virus was upscaled, analyzed via PCR and sequenced as before to ensure the relevant modifications were intact upon viral formation.

**Fig 6:**
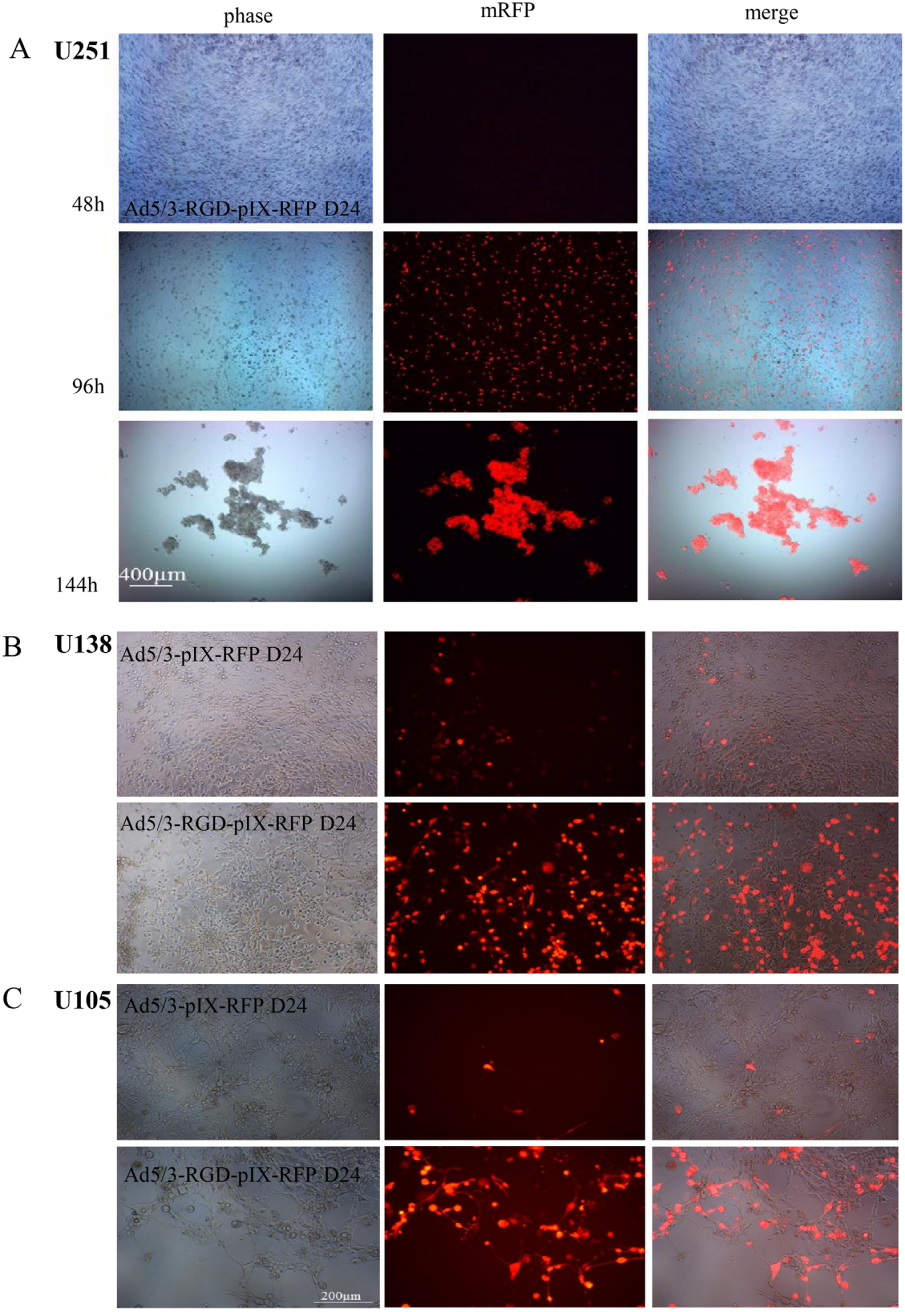
Capsid pIX expression in glioma cells. (A) U251 glioma cells were seeded at 10,000 cells/well and infected the following day at an MOI of 1 VP/cell (Live images taken at 40 × magnification at the times indicated). (B) U138 and (C) U105 cells were infected with Ad5/3-IX-RFP D24 (top panels) and Ad5/3-RGD-IX-RFP D24 (lower panels) at an MOI of 1 VP/cell for six days and at an MOI of 10 VP/cell for nine days, respectively (100 × magnification live images in a 96 well).

The new CRAd, Ad5/3-RGD-IX-RFP D24, was then tested in the glioma line U251 to see if it maintained CPE while expressing pIX incorporated proteins. Monitoring pIX-RFP expression in infected U251 glioma lines indicated that the protein increased over time in a viral DNA replication dependent manner (Fig 6A).

**Fig 7:**
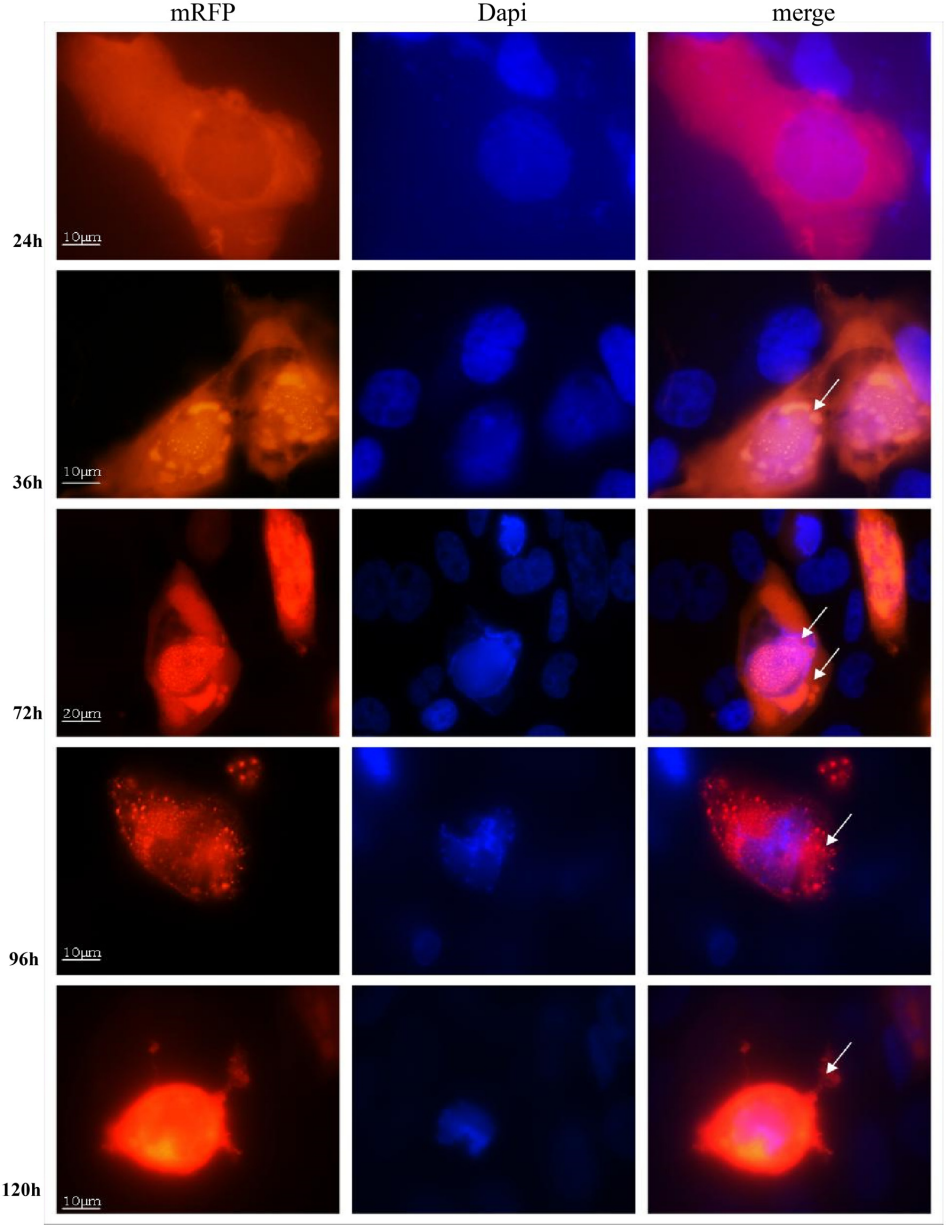
Capsid pIX intracellular expression. U251 cells were infected at an MOI of 1 VP/cell and then fixed at the times indicated with PBS containing 10% formalin, and mounted with Vectashield with DAPI. Fluorescent signal for pIX-RFP was detected by fluorescent microscopy (all magnifications at 1,000 × except 72h at 600 ×). Arrows indicate virus particle specs in the cytoplasm by 24 hpi, in the nucleus and vacuoles by 36 hpi; viral specs released from the nucleus into the cytoplasm by 96 hpi, and released from the cell by 120 hpi.

To ensure the virus maintained enhanced targeting and oncolysis, Ad5/3-RGD-IX-RFP D24 was compared with a previously generated Ad5/3-IX-RFP D24 [34]. Similar to the improvement in oncolysis by the Ad5/3-C-RGD D24 over the unmodified Ad5/3 D24, an increase in the number of fluorescent cells was detected in the RGD modified fluorescent oncolytic virus (Fig 6B and 6C and S6 Fig). The fluorescent labeling technique also allowed accurate observation of unique aspects of viral replication and the fate of pIX expressed proteins. Importantly, analysis of viral and protein intracellular trafficking indicates that both the multi-targeting modifications and pIX protein expression did not adversely affect viral infection, trafficking or release as the time course were similar to reports from first and second generation viruses: preferential localization in the cytoplasm at 24 hours post-infection (hpi) and nuclear localization by 36 hours, indicative of the necessity of the protein in virus particle assembly [25]. Large vacuoles, characteristic of autophagy, could be seen in the infected cells by 72 hpi similar to reports from other D24 virus infected cells [35]. Nuclear membrane integrity appeared fragile with virus release from the nucleus by 96 hpi followed by release from the cell by 120 hpi (Fig 7). The increase in expression of RFP as the virus replicates is an important observation suggesting that any neoantigen expression will be enhanced as the multi-targeting virus replicates improving the potential that antigen presenting cells (APC) will recognize the TSAs.

Hence, the data demonstrates that the large globular protein RFP is functional at this site while the virus maintains enhanced oncolysis over the Ad5/3-RFP D24. The data further suggests that this capsid protein can be used for the attachment of TSAs and that the expression of these antigens will be enhanced as seen in the enhancement of RFP signals over the second generation CRAd. Furthermore, the Ad5/3-RGD-IX-RFP D24 may provide important imaging data for detecting, tracking and monitoring both the spread of the virus and infected tumor cells.

### E3 expression of immunotherapeutic agents

OV delivery of immunotherapeutic drugs such as cytokines, checkpoint inhibitors, and prodrugs is an ideal way to induce sustained expression of the agents safely to local tumor tissue. Hence, Ad5/3-C-RGD D24 was engineered to ascertain if the virus could express drugs that may aid an immunotherapeutic response while maintaining enhanced heterogenic oncolysis. Construction of the new virus was initiated by engineering a number of modified shuttle vectors (SV). Similar to the one made above, these vectors contain recombination arms with AdezDF-swa1; and the fiber was modified to incorporate the 5 shaft with the Ad3 serotype knob followed by the RGD modification on the C-terminus. Along with adding antibiotic resistance to aid in HR, a large portion of the E3 gene was deleted and replaced with unique restriction sites *BstBI* and *SalI* for cloning of immunostimulatory agents. Expression of genes cloned into this site are under control of the immediate early gene E1A [36]. Importantly, the adenovirus death protein was included in the shuttle vector as overexpression of this protein has shown enhanced oncolysis and viral spread [37]. Furthermore, to aid an immune response a number of genes that down regulate the immune response such as E319k have been deleted. This gene serves a similar function as the ICP47 gene in HSV which was deleted in T-Vec. Both of these proteins bind and retain MHC Class I proteins preventing viral antigen presentation [38, 39]. Hence, by deleting these regions, Ad5/3-C-RGD D24 infected cancer cells may be less likely to suppress an immune response to those cells potentially enhancing APC activity and TSA processing.

A number of variations of the SVs were made until a successful vector was formed that proved compatible with rescuing the virus while maintaining the enhancement in cytotoxicity. Double homologous recombination steps were carried out as before (Fig 5).

Following the successful rescue of Ad5/3-RGD E3 D24, a new shuttle vector incorporating the cytokine GM-CSF was generated by inserting the immunostimulant into the unique *BstBI* and *SalI* sites generated during the construction of the above SVs. Granulocyte-macrophage colony stimulating factor (GM-CSF) is a potent inducer of antitumor immunity, activating natural killer cells, DCs and cytotoxic CD8+ T-lymphocytes. However, when GM-CSF is used systemically, the immune stimulant is compromised by toxic side effects and limited efficacy due to poor delivery to tumors [40]. Hence, by utilizing Ad5/3-C-RGD D24, GM-CSF should be delivered deep into the tumor, producing both a debris field rich in tumor epitopes, costimulatory danger signals along with enhanced cytokine signaling. The goal of the multiple simultaneous treatments being to bring about a consistent immune response targeted to the tumor antigens efficacious enough to halt or eliminate the cancer.

**Fig 8:**
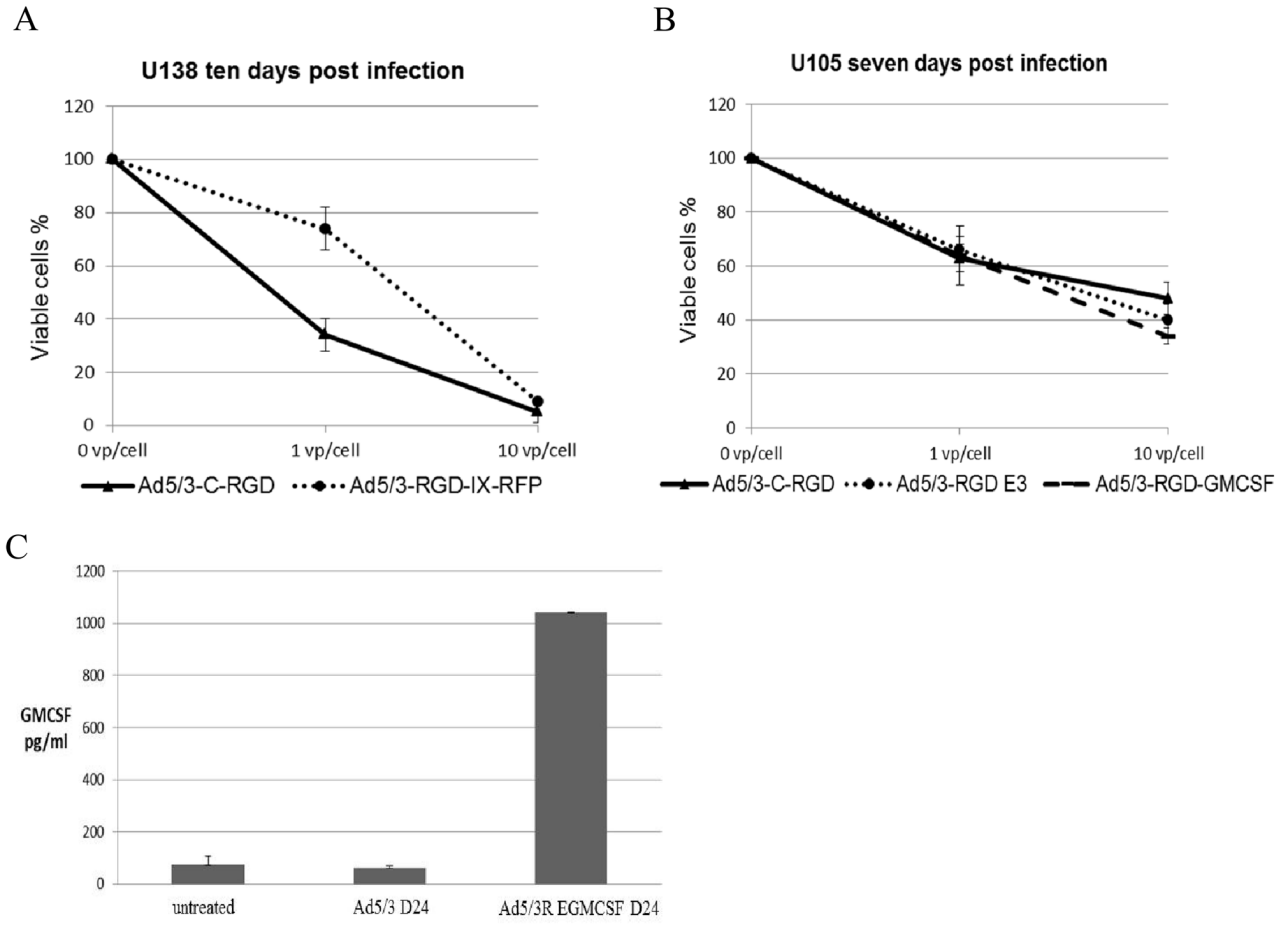
Oncolysis and drug delivery expression of the multi-targeting virus platform. Examples of cancer killing between the vaccine platform Ad5/3-RGD-IX-RFP D24 and the unmodified version (A), and between the E3 modified virus with and without GM-CSF expression (B). (C) GM-CSF production in A549 cells. Similar results were seen in other cell lines assayed. All experiments were carried out in triplicate, three or more times. Data presented as mean ± standard deviation.

Upon successfully generating the E3-GMCSF 5/3-RGD SV the complete genome was HR, rescued and upscaled as above (Fig 5). The new virus showed similar oncolysis as the non-drug delivery viruses and produced over 1000pg/ml of GM-CSF at low titer (10VP/cell) only 48 hpi as detected by ELISA (Fig 8).

## Discussion

Oncolytic viruses show therapeutic potential in treating a range of cancers. In this study the promising Ad5/3 D24 oncolytic adenovirus [5, 12, 26, 41–44] has been further modified by incorporating an RGD motif onto the C-terminus of the Ad3 knob. The modification resulted in equal to, or improved in vitro cell killing in all cancer lines assayed when compared to the cytotoxicities of Ad5 D24, Ad5-RGD D24, Ad5/3 D24 or the HSV M002 viruses. The enhancement was most pronounced at low titers.

The Ad5/3-C-RGD D24 virus targets multiple receptors upregulated on cancer cells: integrins and the receptors for Ad3. All cell lines tested were positive for αVβ3, αVβ5 integrins or DSG2 a primary receptor for Ad3. Hence, this multi-targeting CRAd may have potential to target a wide range of cancers as full CPE has been detected in all cancer lines assayed to date including glioma, ovarian, breast, pancreatic, prostate, lung and neuroblastomas.

The first focus of this study was to improve oncolysis of the Ad5/3 D24 CRAd. The Ad5/3 component of this virus has numerous therapeutic benefits: first, the modification redirects targeting to receptors up regulated on a number of cancers and away from CAR, the primary receptor for Ad5, which is down regulated on many tumor types while being expressed on normal cells like those of the liver and lung [45]. Also DSG2, a primary receptor for Ad5/3, is a component of the epithelial cell-cell adhesion structure [14, 15]; hence, directing OVs to this receptor may greatly aid drug delivery, as binding of the virus to DSG2 triggers the opening of tight junctions. By improving the targeting capacity of Ad5/3 with the RGD modification, the opening of tight junctions may further enhance access to other cancer receptors allowing improved delivery of the virus, and therapeutic agents expressed by the virus, deep into the tumor. This enhancement in oncolysis and delivery should improve the interaction of immune-related inflammatory cells with TSAs generated in the debris field of infected cancer cells. Furthermore, such a modification may enhance conventional radiation and chemotherapy treatments when paired with the virus, as the Ad5/3 component of the virus has shown to improve access to the tumor microenvironment.

Previous reports indicate that the receptors for Ad3 are up-regulated on a number of cancer lines, whether these receptors consist of DSG2 or other receptors is unclear. The analysis here provides evidence of the importance of DSG2 in Ad5/3 oncolysis: the glioma lines U105 and U138 expressed little to no DSG2. In the U105 line, the result was little to no oncolysis for Ad5/3 D24 while the multi-targeting CRAd showed full CPE. Full CPE was also seen in U138 cell lines treated with Ad5/3-C-RGD D24. Interestingly, given the lack of DSG2, Ad5/3 D24 though attenuated did result in full CPE at the higher titers in the U138 line suggesting the virus may be accessing other receptors besides DSG2 that are not present on the U105 cell line.

Regardless, the data here indicate that Ad5/3 D24 has limited infectivity in a number of cancer lines at low titers (e.g. 1VP/cell): glioma, breast, ovarian and neuroblastoma lines. The same lack of infection of certain cancer cells can be seen with the Ad5-RGD D24 and the HSV OVs. However, by targeting multiple receptors, a synergistic effect seems to occur in which the multi-targeting virus displays enhanced oncolysis even if integrins and/or DSG2 are not expressed or weakly expressed.

The improved cytotoxicity may simply be due to an increase in the number and variety of receptors accessible, aiding infection most when the VP/cell ratio is low. A synergistic effect could also be the result of the increase in the RGD-integrin interaction combined with the activation of DSG2. The former has been shown to improve infection by enhancing the signaling cascade responsible for internalizing the virus [46] while the later opens tight junctions improving access to other receptors on tumor cells aiding oncolysis and viral spread [47]. Future work will look into these possibilities and how the multi-targeting strategy is resulting in such a synergistic enhancement in oncolysis. Nonetheless, given that tumors are extremely heterogenic and the obvious need to target as many cancer cells as possible with the fewest virus particles prior to Ad clearance, the Ad5/3-C-RGD D24 should produce a marked therapeutic improvement.

Recent clinical data indicates that successful OV treatment for cancer patients depends on triggering systemic antitumor immune responses. The immune system plays a key role in cancer development and progression: it can suppress tumor growth but it can also select for tumor cells facilitating tumor outgrowth. Immunotherapy centers on augmenting the immune response in four ways: first, enhancing the immune effector process against cancer by activating lymphocytes and cytokines; second, developing vaccines to elicit specific immune responses to tumor antigens; third, using antibodies to target and eliminate tumors; fourth, inhibiting cellular mediators that induce cancer immunosuppression. Ad5/3-C-RGD D24 has potential to be used in all four approaches. Adenovirus infected cancer cells trigger both an innate and adaptive immune response. Also, the Ad5/3-C-RGD D24 is able to infect and kill all cancer lines assayed to date at low titers, which should create a larger debris field of potential TAAs, danger signals and cytokines in a more diverse population of cancer cells. Despite the heterogenic oncolytic enhancement, to generate a consistent complete response in cancer patients, the virus will most likely need to be timed with existing therapies and deliver therapeutic agents to optimize the immune response, as there is no certainty that adequate TSAs will be released and or the danger signals may lack robust efficacy to circumvent immunosuppression regardless of the larger debris field.

To further optimize the therapeutic treatment of the virus, Ad5/3-C-RGD D24 was engineered to deliver both potential TSAs and immunotherapeutic drugs. As deep sequencing combined with other techniques are discovering immunogenic mutant peptides that can serve as T-cell epitopes, Ad5/3-C-RGD D24 was first tested to determine if the capsid could be a site for attachment for such TSAs in a future OV vaccine strategy. For proof of principle, RFP was successfully cloned onto the capsid protein pIX. The data demonstrates that the large globular protein is functional at this site. Ad5/3-RGD IX-RFP D24 not only displayed enhanced cancer killing but also expressed more potential antigens over the second generation CRAd.

The Ad5/3-RGD-IX-RFP D24 virus will also serve as a readout of viral replication and accumulation in tumors, as well as a control for delivery of the agents immediately or shortly after administration, i.e. prior to the onset of reporter gene expression and CRAd replication. Of note, the pIX RFP modified virus did show a decrease in oncolysis compared to the unmodified Ad5/3-C-RGD D24. The most likely explanation for this is that the infectious titer was carried out via VP/ml, which underestimates the RFP virus as the RFP protein skews the OD260 reading. In other words, the OD260 measures RFP as virus particles [25]; hence, fewer virus particles were added.

Next, to aid delivery of immunotherapeutic agents, a number of novel shuttle vectors were engineered and through an extensive trial and error process various modifications were made to the vectors until viable viruses were generated that were capable of drug delivery while maintaining the improved oncolysis. The adenovirus death protein was incorporated into this genome while a number of genes that down regulate the immune system have been removed to maximize both oncolysis and an immune response. Importantly, no decrease in cancer killing has been detected in the drug delivery E3 modified viruses with similar CPE seen in all cancer lines assayed along with GM-CSF production. With this platform, new armed Ad5/3-C-RGD D24 viruses can be made, upscaled and ready for use in as little as three months, raising the possibility of enhanced heterogenic oncolysis while delivering TSAs, monoclonal antibodies, checkpoint inhibitors, cytokines, and other agents tailored to specific patient cancer types with the goal of augmenting an immune response capable of halting or eliminating the cancer.

Obviously much work needs to be done to ascertain if the improvement in cancer killing also occurs in vivo and if an effective immune response is generated with the various multi-targeting viruses. Unfortunately, immunocompetent animal models for adenovirus are wanting as Ad does not replicate normally in animal cells other than human, possible due to translational impairment [48]. Nonetheless, there have been reports that certain mouse cell lines are permissible to Ad replication. For preliminary work, the metastatic breast cancer mouse line 4T1 was assayed for adenovirus infection. Although Ad5/3-C-RGD D24 was attenuated in the cell line it did display oncolysis and production of GM-CSF at high titers (S7 Fig) raising the possibility that immunotherapeutic drug delivery in this metastatic model could be tested. Hence, future work will focus on in vivo models, studies on viral spread, synergies of multi-targeting, and most importantly arming and pairing Ad5/3-C-RGD D24 with adjunctive treatments to enhance an immune response against a variety of metastatic cancers.

## Supporting Information

S1 Fig Cell killing assays in glioma cell lines. Ad5 D24, Ad5/3 D24 and Ad5/3-C-RGD D24 were compared in the glioma lines D54, U138 and U105. Cell viability was measured by MTS assay on the days indicated as previously described. Data presented as mean ± standard deviation.
(TIF)

S2 Fig Cell killing assay in human fibroblast cells. (A) Ad5/3 D24 and Ad5/3-C-RGD D24 were compared in the normal cell line HFF. Cell viability was measured by MTS assay five days post infection. Error bars represent standard deviations. (B) Live images in a 96 well plate prior to MTS assays (100 × magnification)
(TIF)

S3 Fig Phase contrast images of Ad5/3 D24 and Ad5/3-C-RGD D24 in D54-MG (glioma) six days post infection; OV-4 (ovarian) nine days post infection; DU145 (prostate) and A549 (lung) six days post infection (All at an MOI of 1VP/cell and 100 × magnification live images in a well of a 96 well plate). (TIF)

S4 Fig Release of Ad5/3-C-RGD D24 from infected cells to culture medium. SK-N-As and -Be cells were infected with Ad5/3-C-RGD D24 at an MOI of 1 PFU/cell. Culture medium containing released virus particles from the infected cells was harvested at the indicated time points. Infectious titers from each time point were measured by TCID50 on 549 cells. Error bars represent standard deviations.
(TIF)

S5 Fig Phase contrast images of neuroblastoma lines treated with multiple OVs. SK-N-As and Be cells were infected with the indicated OVs at an MOI of 0.1 or 1.0 PFUs/cell. Live images at 60 hpi in a 96 well plate prior to MTS assays (100 × magnification).
(TIF)

S6 Fig Enhanced protein expression by Ad5/3-C-RGD-IX-RFP D24. U138 and U105 cells were infected with Ad5/3-IX-RFP D24 and Ad5/3-RGD-IX-RFP D24 at an MOI of 1 VP/cell for six days and at an MOI of 10 VP/cell for nine days, respectively. Percentage of cells displaying fluorescence was measured in quadruplet as previously described [25]. Data presented as mean ± standard deviation.
(TIF)

S7 Fig Ad5/3-C-RGD D24 oncolysis and GM-CSF production in a metastatic breast cancer mouse line. A) Ad5/3 D24 and Ad5/3-C-RGD D24 were compared in the mouse breast cancer cell line 4T1. Cell viability was measured by MTS assay as previously described. B) GM-CSF production by Ad5/3-RGD E3 GMCSF D24 in mouse cell line 4T1 seven days post infection.
(TIF)

S1 Table VP/ml titers of CRAds used in this study. * indicate novel oncolytic viruses generated in this study.
(TIF)

S2 Table PFU/ml titers of oncolytic viruses used in this study. * indicate novel oncolytic viruses generated in this study.
(TIF)
