# Supplementary figures and images for: A Multi Targeting Conditionally Replicating Adenovirus Displays Enhanced Oncolysis while Maintaining Expression of Immunotherapeutic Agents
Source: PLoS One. 2015 Dec 21;10(12):e0145272. doi: 10.1371/journal.pone.0145272 (PMC4687127; doi:10.1371/journal.pone.0145272)

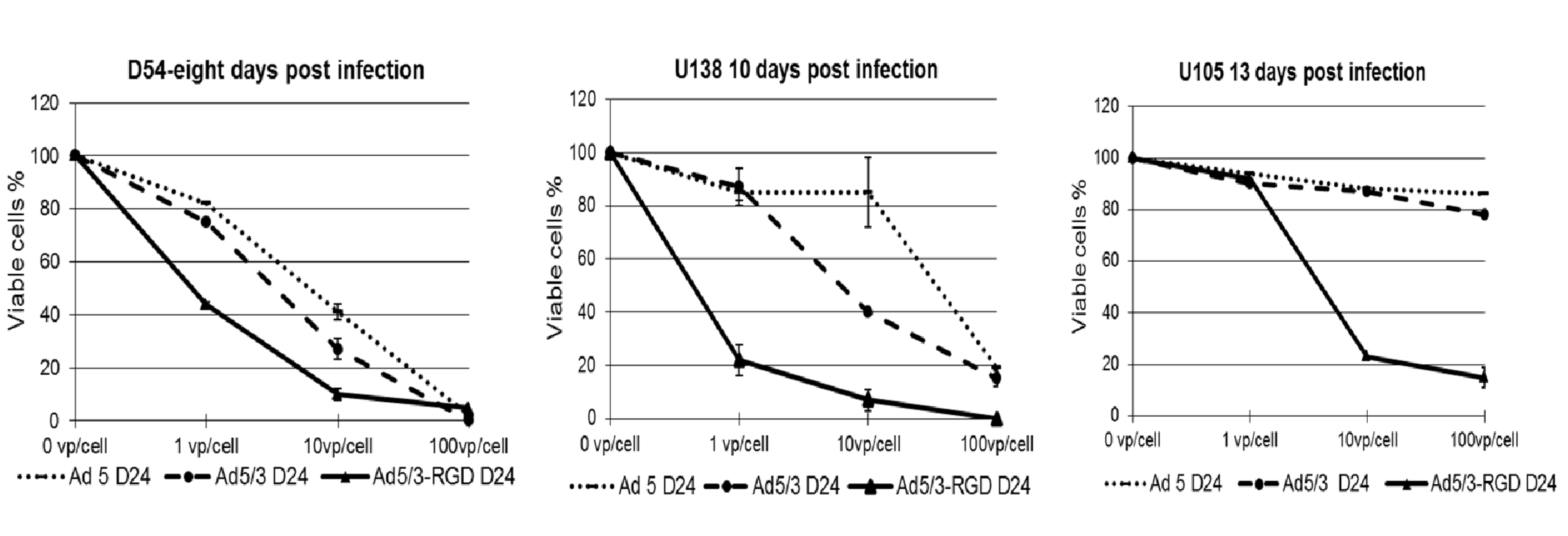

Supplement: S1 Fig — Ad5 D24, Ad5/3 D24 and Ad5/3-C-RGD D24 were compared in the glioma lines D54, U138 and U105. Cell viability was measured by MTS assay on the days indicated as previously described. Data presented as mean ± standard deviation. (TIF) [file pone.0145272.s001.tif]

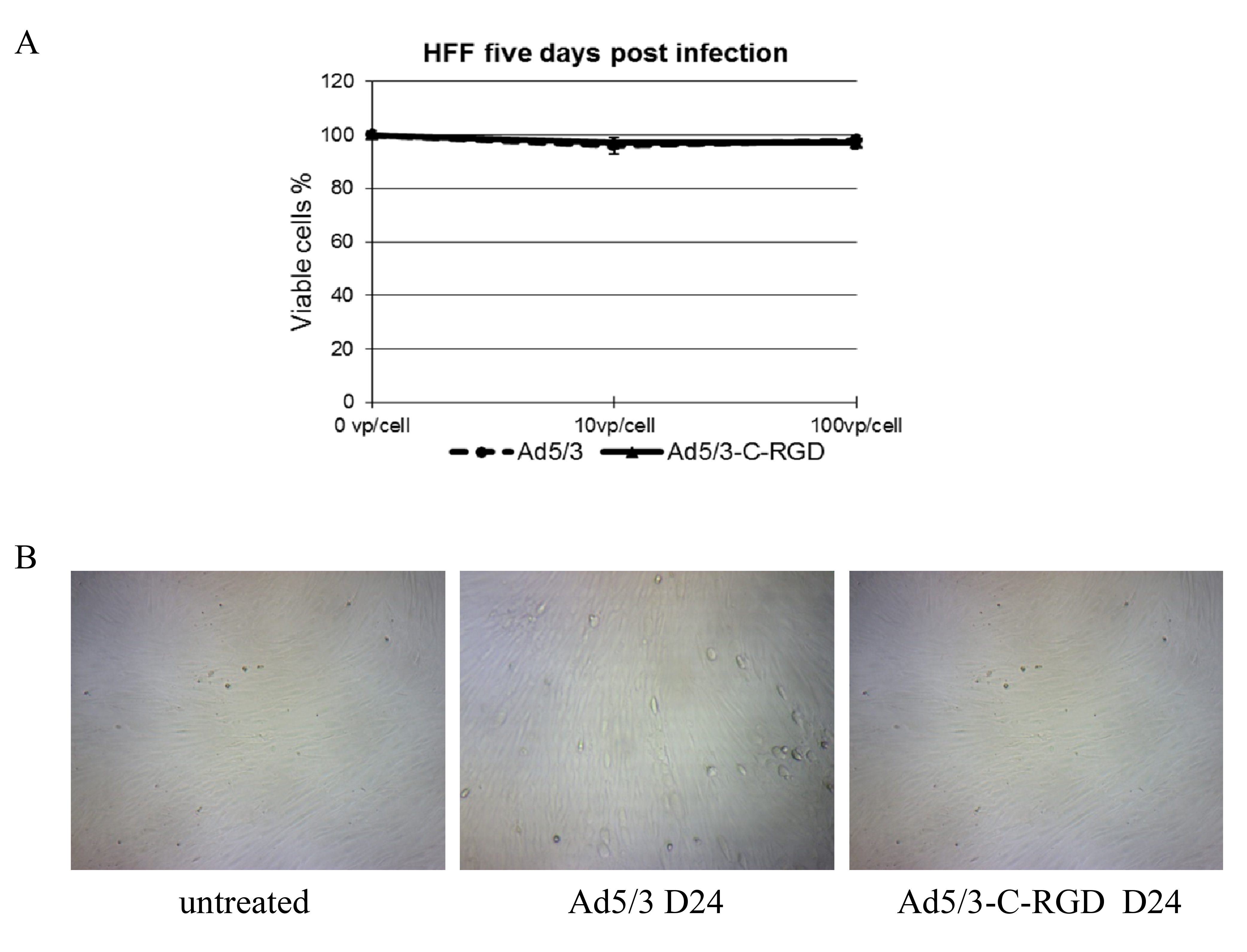

Supplement: S2 Fig — (A) Ad5/3 D24 and Ad5/3-C-RGD D24 were compared in the normal cell line HFF. Cell viability was measured by MTS assay five days post infection. Error bars represent standard deviations. (B) Live images in a 96 well plate prior to MTS assays (100 × magnification) (TIF) [file pone.0145272.s002.tif]

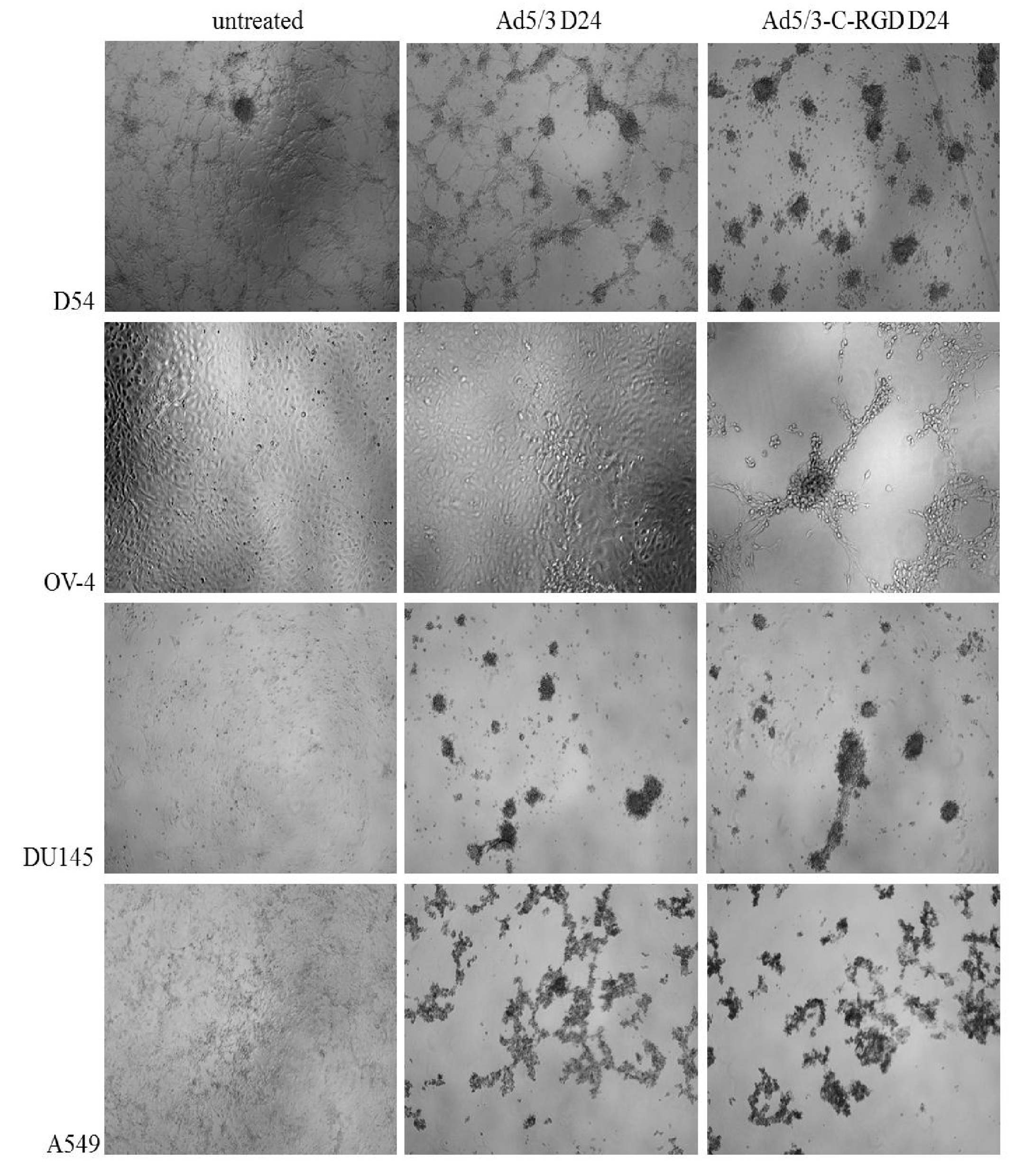

Supplement: S3 Fig — (TIF) [file pone.0145272.s003.tif]

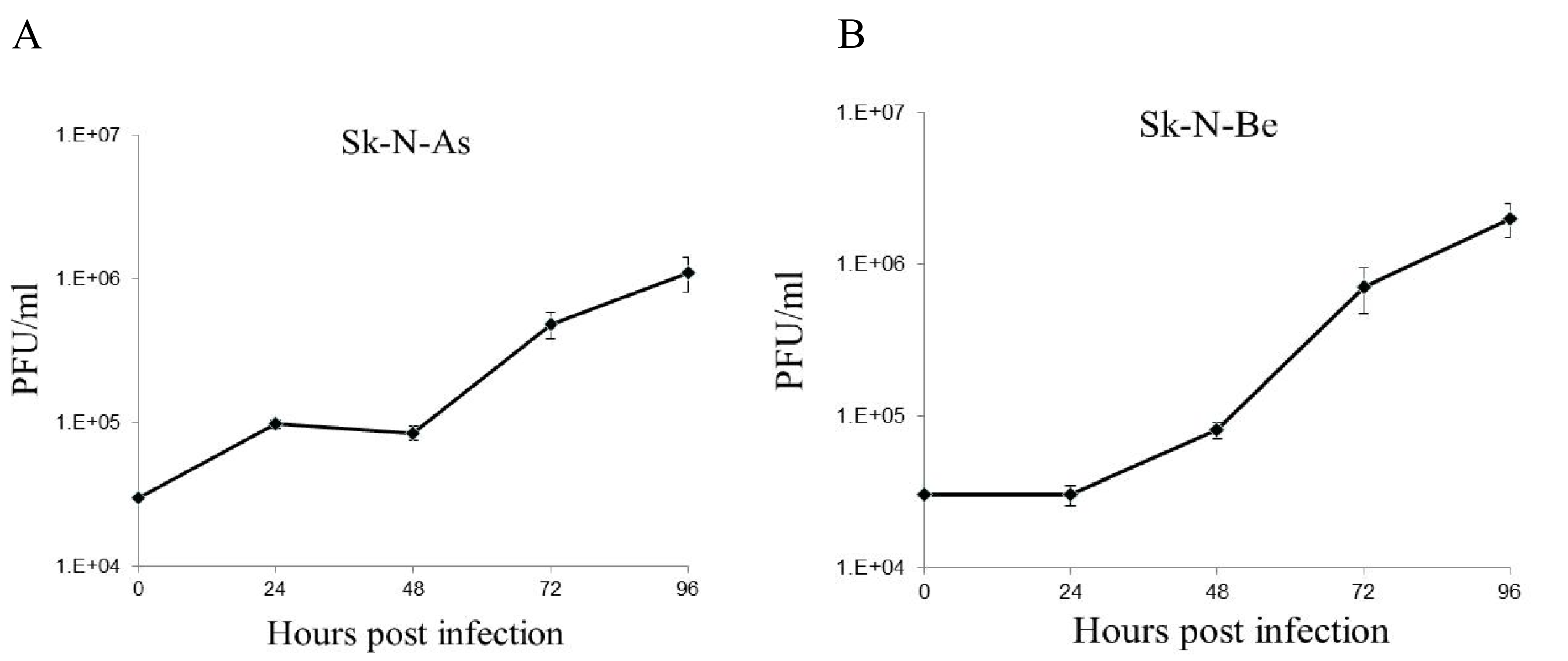

Supplement: S4 Fig — SK-N-As and -Be cells were infected with Ad5/3-C-RGD D24 at an MOI of 1 PFU/cell. Culture medium containing released virus particles from the infected cells was harvested at the indicated time points. Infectious titers from each time point were measured by TCID50 on 549 cells. Error bars represent standard deviations. (TIF) [file pone.0145272.s004.tif]

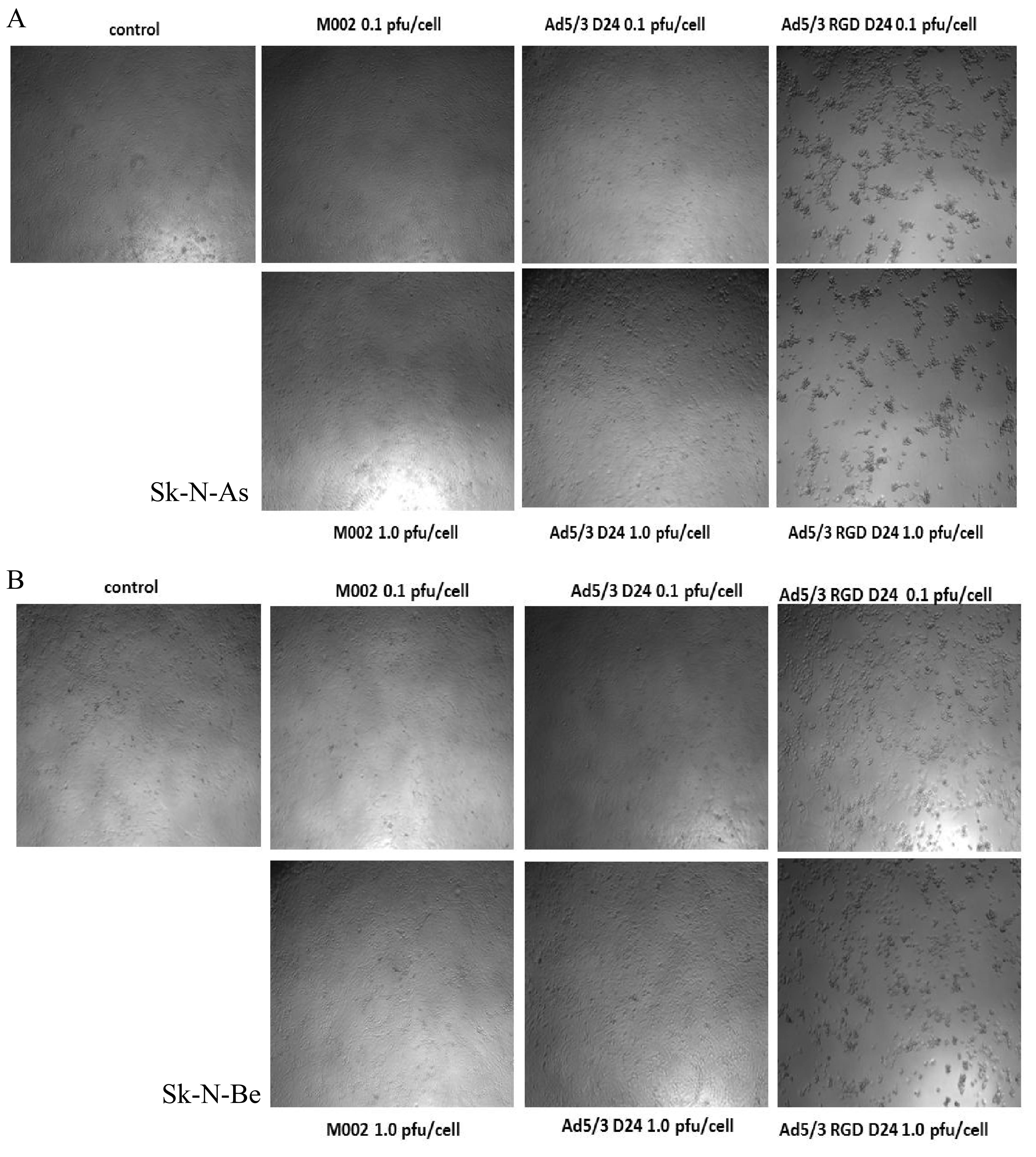

Supplement: S5 Fig — SK-N-As and Be cells were infected with the indicated OVs at an MOI of 0.1 or 1.0 PFUs/cell. Live images at 60 hpi in a 96 well plate prior to MTS assays (100 × magnification). (TIF) [file pone.0145272.s005.tif]

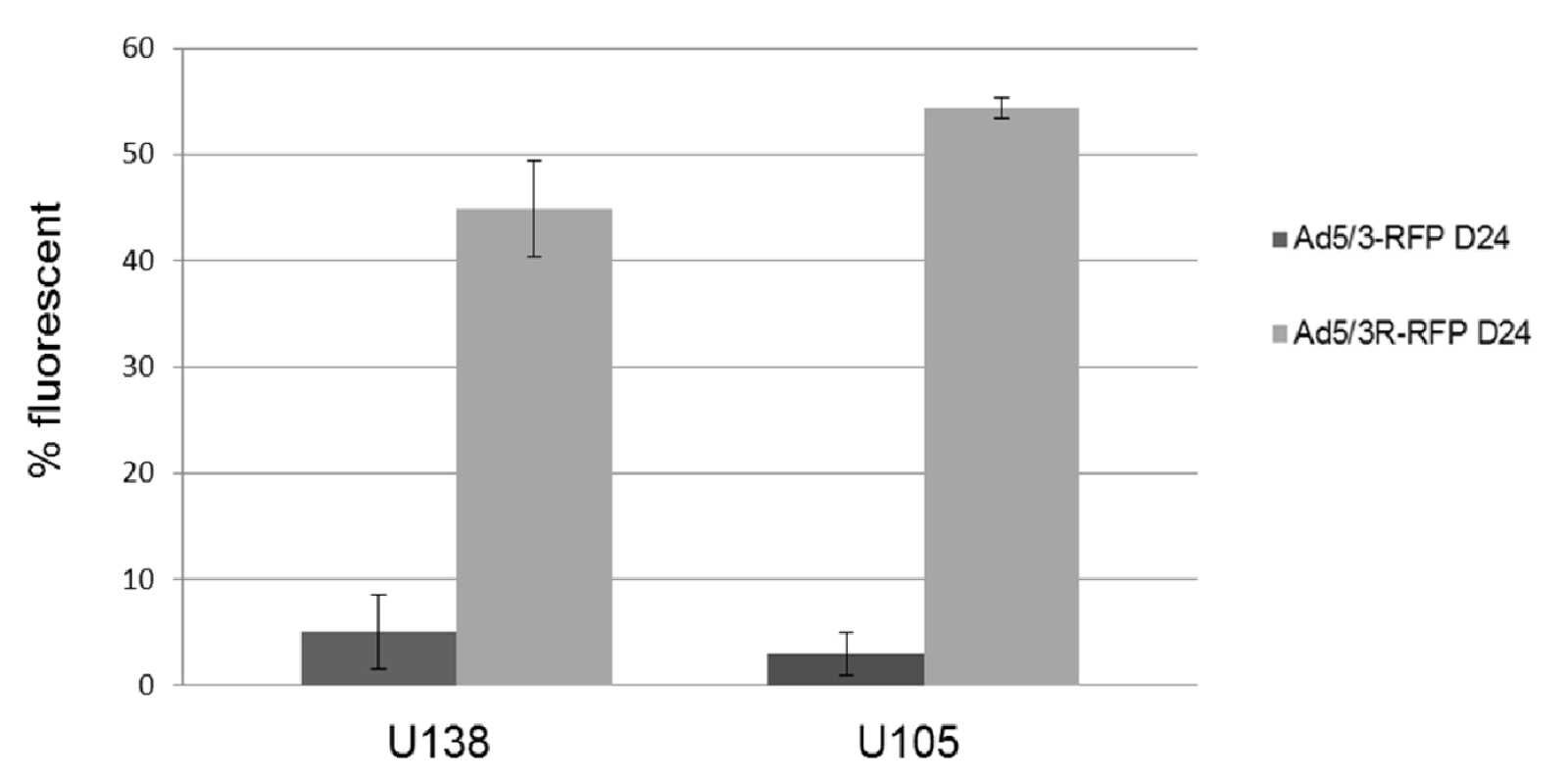

Supplement: S6 Fig — U138 and U105 cells were infected with Ad5/3-IX-RFP D24 and Ad5/3-RGD-IX-RFP D24 at an MOI of 1 VP/cell for six days and at an MOI of 10 VP/cell for nine days, respectively. Percentage of cells displaying fluorescence was measured in quadruplet as previously described [25]. Data presented as mean ± standard deviation. (TIF) [file pone.0145272.s006.tif]

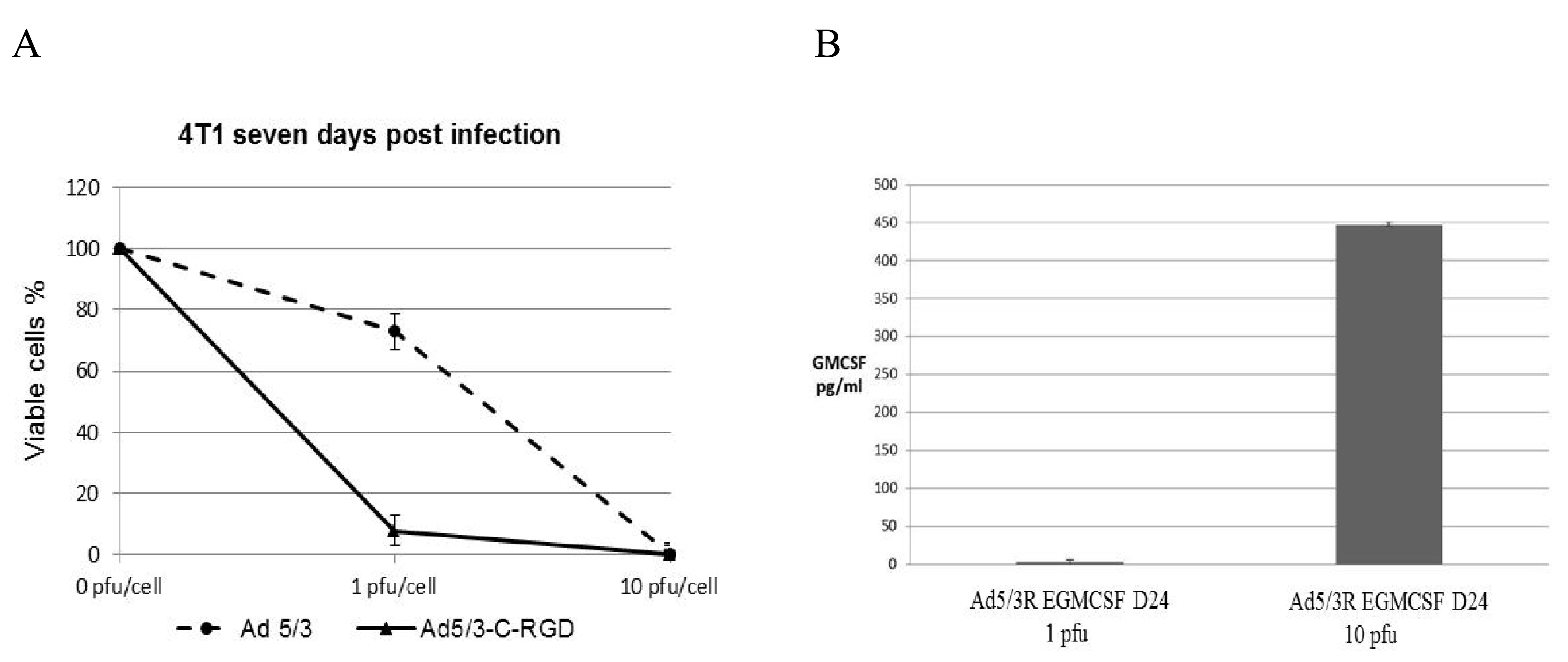

Supplement: S7 Fig — A) Ad5/3 D24 and Ad5/3-C-RGD D24 were compared in the mouse breast cancer cell line 4T1. Cell viability was measured by MTS assay as previously described. B) GM-CSF production by Ad5/3-RGD E3 GMCSF D24 in mouse cell line 4T1 seven days post infection. (TIF) [file pone.0145272.s007.tif]

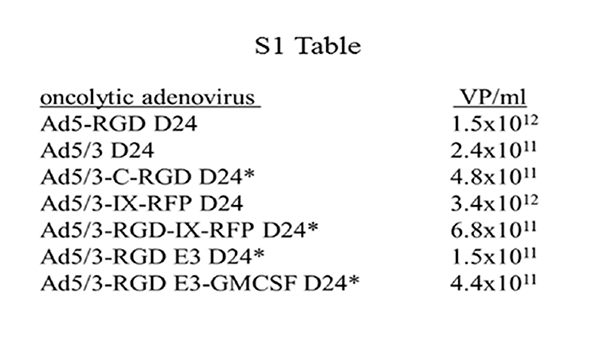

Supplement: S1 Table — * indicate novel oncolytic viruses generated in this study. (TIF) [file pone.0145272.s008.tif]

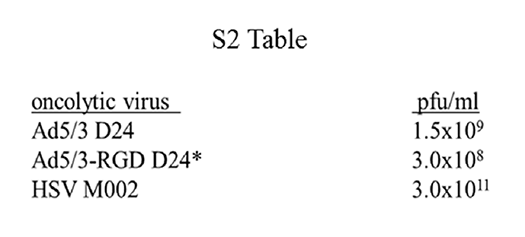

Supplement: S2 Table — * indicate novel oncolytic viruses generated in this study. (TIF) [file pone.0145272.s009.tif]
